# Clinical Lessons on Nervous Diseases

**Published:** 1898-04

**Authors:** 


CLINICAL LESSONS ON NERVOUS DISEASES—By S. Weir Mitchell, M. D., L. L. D., Edin; Member of the National Academy of Sciences; Honorary Fellow of the Medico-Chirurgical Society of London. Published by Lea Bros. & Co., Philadelphia and New York. Cloth, $2.50.

There are many men and but few masters, of whom, since the death of the late lamented Charcot, if not before, S. Weir Mitchell is head master in his department. The little book of 305 pages is priceless, from the fact that it is a part of the clinical experience of this, the greatest clinician of his time. It is conveniently divided into chapters, each one devoted to some of the rarer forms of nervous disease, and each disease illustrated by actual cases from his apparently inexhaustible case-book. The writer sent him a case a few years since which baffled the medical skill of an entire section of the State; a man of great usefulness in his community, stricken a helpless and apparently hopeless invalid in the prime of life. In a few months he returned completely restored, and it looked like magic to us who had treated him in vain. The case is reported in this little book, and it is like a revelatson to read his clear analysis of the conditions of the patient, and the unique manner by which he reached a clear solution of condition and successful treatment. If you will buy this little book, you will not want to lay it down until you have finished reading it. Some of the titles which are suggestive are as follows: Psychic Blindness, Seasonable Melancholia, Some of the Disorders of Sleep, Hysterical Myoclonus, Subjective Sensations of Heat and Cold, etc. etc. J.

